# A Thermoelectric Generator Using Porous Si Thermal Isolation

**DOI:** 10.3390/s131013596

**Published:** 2013-10-10

**Authors:** Emmanouel Hourdakis, Androula G. Nassiopoulou

**Affiliations:** NCSR Demokritos/IMEL, Terma Patriarchou Gregoriou, Aghia Paraskevi, Athens 15310, Greece; E-Mail: mhour@imel.demokritos.gr

**Keywords:** thermoelectric generator, porous Si, thermal conductivity

## Abstract

In this paper we report on a thermoelectric generator (TEG) using thermal isolation provided by a thick porous Si layer locally formed on the Si wafer and thermocouples composed of p-doped polycrystalline Si/Al. The “hot” contacts of the thermocouples lie on the porous Si layer, while the “cold” contacts lie on bulk crystalline Si. A housing was also designed and fabricated in order to transfer any external temperature change on the “hot” contacts of the thermocouples, the “cold” contacts being isolated from the “hot” contacts by a thick resist layer. The fabrication of the sensing element (Si die) is fully compatible with batch Si processing. The output power of the thermoelectric generator depends on the porous Si isolation layer thickness, porosity, structure and morphology. For a mesoporous Si layer of 60% porosity and a macroscopic temperature differential of 10 K, an output power of 0.39 μW/cm^2^ was measured for a 50 μm thick porous Si layer.

## 1. Introduction

Solid state thermoelectric generators (TEGs) are devices that can convert thermal gradients to electrical power through the Seebeck effect [1]. These devices are reliable, low noise and low cost, but suffer from low conversion efficiencies [2,3]. On the other hand, energy is wasted in the form of heat for a vast variety of everyday situations, ranging from exhaust pipes in cars, to heat generated on a microprocessor to even heat generated by the human body. Any device that can transform that heat into electrical power could potentially be very useful, provided that the obtained power is sufficient for practical applications. An effort is so needed in order to increase the output power of any thermoelectric generator. On the other hand, the continuous power reduction of state-of-the-art electronics, for example wireless devices, makes thermoelectric devices closer to practical applications.

The two factors that determine the merit of a specific thermoelectric generator in terms of practical applications are the conversion efficiency and the cost. The conversion efficiency depends both on the materials used and the device structure. Commercial state-of-the-art thermoelectric generators use materials with high efficiency such as alloys of Bi, Te, Sb and Se which possess dimensionless figures of merit close to 1 [4]. These materials though cannot be processed using CMOS techniques and are therefore cost-intensive. Si batch processing is a means of ensuring low cost devices, as these processes are well established and can produce large numbers of devices with small surface areas. So, even though materials compatible with Si processing such as polycrystalline Si and SiGe have small dimensionless figures of merit, they have attracted attention for use in thermoelectric generators as they can significantly lower the cost. Almost all the examples in the literature using poly-Si or poly-SiGe thermocouples use suspended membranes created by MEMS techniques [5–13] to thermally isolate their hot contacts from the cold contacts, these last being integrated on the Si substrate. However, although the free standing membranes constitute an excellent thermal isolation platform [5–10], their fabrication scheme is relatively complicated and large area membranes lack mechanical stability and compatibility with standard Si processing. In addition, in some cases wafer bonding is needed in order to assure thermal contact to a heat source at the top [7,9] or the bottom [5] surface of the initial substrate. The use of this additional Si wafer increases the cost of the devices.

Bulk crystalline Si has long being considered as an inefficient thermoelectric material with a figure of merit ZT ≈ 0.01, due to its high thermal conductivity. However, much higher values can be achieved by Si nanostructuring (using Si nanowires [14] or nanostructured porous Si [15]). This is attributed to a significant decrease in material thermal conductivity due to phonon confinement in nanostructures. This has led to attempts to incorporate such nanostructures in a Si batch process for thermoelectric generators [16]. However, the fabrication complexity, combined with the large heat resistance of the contact to such structures, is still challenging. Very promising are the very recent results in ref. [17], demonstrating that heavily boron-doped polycrystalline Si material with grain sizes ≈ 30 nm can reach a ZT ≈ 0.65, value that is interesting for practical applications. In this material, both the thermal conductivity is decreased by nanostructuring and the electrical conductivity is increased by the high doping concentration. This result opens new possibilities in the use of polycrystalline Si, a material widely used in Si technology and fully compatible with batch Si processing, in thermoelectrics.

In this work we propose a thermoelectric generator using standard boron-doped polycrystalline Si/Al thermocouples, and thermal isolation of the active device by a thick highly porous nanostructured Si layer. Porous Si is a versatile material with tunable properties that depend on its structure and porosity [18–20]. It shows very low thermal conductivity (more than two orders of magnitude lower than that of bulk crystalline Si at room temperature) [21–23] that provides effective thermal isolation from the Si wafer [23], without using any free standing parts. It is locally formed on the Si wafer and its surface is co-planar to the rest of the Si surface. It has been successfully used as an isolation platform on the Si substrate in a large number of Si thermal sensors and other applications [24–28]. The thermoelectric generator presented in this work is shown to produce comparable output power and open-circuit voltage as thermoelectric generators using free standing membranes. It has the additional advantage of using a simple, low cost fabrication process that does not need any Si backside etching or wafer bonding to a solid substrate to assure thermal contact. It is also mechanically very stable. To the best of our knowledge, the use of porous Si thermal isolation in a thermoelectric generator has not been reported before.

## 2. Thermoelectric Generator Design

Thermoelectric generators are based on the Seebeck effect. Thermocouples made out of two different materials are used to convert a temperature difference between their sides to a voltage difference. A thermocouple made out of materials 1 and 2 will produce a voltage of [6]:

(1) $$V_{th}=(\alpha_{1}-\alpha_{2})\Delta T_{th}$$

where *V_th_* is the voltage produced by one thermocouple, α_1_ and α_2_ are the Seebeck coefficients of materials 1 and 2 respectively and *ΔT_th_* is the temperature difference between the two sides of the thermocouple.

In order to maximize the voltage output of a thermoelectric generator a large number of thermocouples are placed electrically in series. They are also placed thermally in parallel so as to assure the same temperature difference across them. The output voltage of the thermoelectric generator is the sum of the output voltages from each individual thermocouple and is given by [6]:

(2) $$V_{\text{out}}=NV_{th}=N(\alpha_{1}-\alpha_{2})\Delta T_{th}$$

where *N* is the number of thermocouples used. Obviously, by increasing the number *N* of thermocouples, the value of *V_out_* increases proportionally. On the other hand, the value of the total electrical resistance of the generator increases as well, given by [11]:

(3) $$R_{g}=N\left(\rho_{1}\frac{L_{1}}{W_{1}t_{1}}+\rho_{2}\frac{L_{2}}{W_{2}t_{2}}\right)$$

where *L*, *W*, *t* and *ρ* are the length, width, thickness and electrical resistivity of the thermocouple parts and the subscripts 1 and 2 refer to materials 1 and 2 respectively. Contact resistances between different materials are not considered in Equation (3). Since the output power of the generator *P_out_* at a matching electrical load is inversely proportional to R_g_, this has to be taken into account in its optimization. *P_out_* is given by [6]:

(4) $$P_{\text{out}}=\frac{V_{\text{out}}^{2}}{4R_{g}}=\frac{N^{2}{\left(\alpha_{1}-\alpha_{2}\right)}^{2}}{4R_{g}}\Delta T_{th}^{2}$$

In order to compare the output power of generators of different area size operating between different temperature differentials, the power efficiency factor *φ* is defined as [5]:

$$\varphi =\frac{P_{\text{out}}}{S\Delta T_{g}^{2}}$$

where *S* is the surface area of the generator and *ΔT_g_* is the temperature differential across it.

The thermal resistance of the thermocouples (*K_th_*) has two contributions in parallel. The thermal resistance of the material 1 parts (*K_1_*) and the thermal resistance of the material 2 parts (*K_2_*) given by [11]:

(5) $$K_{1}=\frac{1}{N}\left(\frac{L_{1}}{k_{1}W_{1}t_{1}}\right)$$

(6) $$K_{2}=\frac{1}{N}\left(\frac{L_{2}}{k_{2}W_{2}t_{2}}\right)$$

and so:

(7) $$\frac{1}{K_{th}}=\frac{1}{K_{1}}+\frac{1}{K_{2}}$$

where *k_1_* and *k_2_* are the thermal conductivities of materials 1 and 2 respectively.

**Figure 1.**
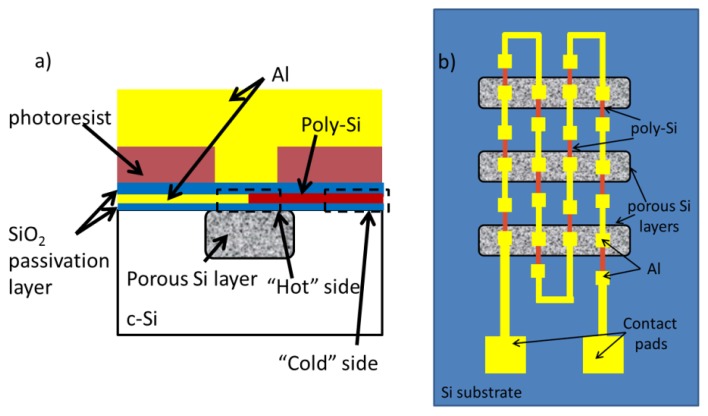
(**a**) Schematic cross sectional view of one part of the thermoelectric generator, illustrating the local porous Si areas, the hot contacts of the thermocouples on top, the patterned photoresist layers that provides thermal isolation between the hot and cold contacts of the thermocouples and the Al thermal bath that transfers the external thermal differences on the hot contacts of the thermocouples; (**b**) Schematic representation of a small number of thermocouples connected electrically in series and thermally in parallel, illustrating the hot contact of the thermocouples on porous Si and their cold contact on bulk crystalline Si. Polysilicon lines are shown in red, while Al lines in yellow.

In this work, we designed and fabricated a simple TEG device with the overall objective to show the effectiveness of thermal isolation by the thick porous Si layer, so the materials and geometry of the thermocouples and the encapsulation materials are at this stage not optimized. The design of the thermoelectric generator consists of 3 different parts, as schematically shown in Figure 1a. The first part is the locally formed porous Si layer on a Si substrate. This layer provides the necessary thermal isolation from the Si substrate, due to its low thermal conductivity (0.23 W/m·K at room temperature, as measured within the authors group for the specific porous Si layer) [23]. A thin SiO_2_ layer is used to provide electrical isolation from the rest of the device. The second part of the design consists of the thermocouples used to convert a temperature difference into electrical power. These thermocouples are composed of p-doped poly-Si and Al. The hot contact of each thermocouple is designed to be on the porous Si layer, while the cold one on the Si substrate (Figure 1a,b). The values of the Seebeck coefficient, the thermal conductivity and the electrical resistivity for the materials used in the thermocouples are presented in Table 1. For the poly-Si material the value of the electrical resistivity was measured using a standard four point probe measurement. The rest of the properties are quoted from a literature report that uses very similar deposition, doping and annealing conditions to the poly-Si used in this work and for which the value of the electrical resistivity is almost the same as in this work. To construct the full generator a large number of these thermocouples are connected electrically in series and thermally in parallel. A schematic example of such a device, showing a small part of the total number of thermocouples, is depicted in Figure 1b. The third part of the design consists of a structure that allows the local heating of the “hot” contact of the thermocouples, lying on the porous Si areas. A thin SiO_2_ layer is first deposited for electrical isolation and then a thick photoresist layer (1.6 μm) is deposited by spinning and patterned so as to define windows on top of the “hot” contacts of the thermocouples for the heat transfer only on the “hot” contacts and thermal isolation of the cold contacts. The photoresist used has a low thermal conductivity (0.19 W/m·K) [29] and it is simple to use in a test device. A thick Al layer was then deposited on top of the whole structure to provide thermal contact to the outside world (see Figure 1).

The thermoelectric generator presented in this work is thus the device that consists of the three parts depicted in Figure 1a. In the designed devices the dimensions of the poly-Si part of the thermocouples were: length L = 55 μm, width W = 10 μm and thickness *t* = 0.5 μm, while for the Al part of the thermocouples they were: length L = 75 μm, width W = 10 μm and thickness *t* = 0.5 μm. The total number of thermocouples was 15,458 and the total device surface area, including contact pads, was 1 cm^2^. Within the total device area, 59 areas with locally grown porous Si layers were designed. Two different series of devices were realized, a first one with 25 μm thick and a second one with 50 μm thick porous Si layers. Since the electrochemical process for porous Si formation is isotropic, the thickness difference translates to a difference in the width of the porous Si layers. Consequently, by using the same mask for the definition of the porous Si surface areas, in the case of the 25 μm thick porous layers the porous Si area width was 105 μm with a 15 μm width of the Si interlayer area, while for the 50 μm thick porous Si layers, their areal width was 115 μm, with 5 μm wide Si layers in between them.

The thermal resistance of a thermoelectric generator as described above can be calculated by considering three thermal resistances in parallel from the top of the generator to its bottom. The first is the thermal resistance of the thermocouples, which is 3.4 K/W using Equations (5–7) and the values of Table 1, the second is the thermal resistance of the porous Si layer and the third is the thermal resistance of the photoresist layer. In the case of the 25 μm thick porous Si layers the total thermal resistance of the generator was calculated at 0.65 K/W, while in the case of the 50 μm thick layers it was 1.22K/W. The values of the thermal conductivity of porous Si and photoresist used are 0.23 W/mK and 0.19W/mK, respectively. Also, the calculated value of the electrical resistance of the thermocouples was 3.76 MΩ in both cases.

**Figure 2.**
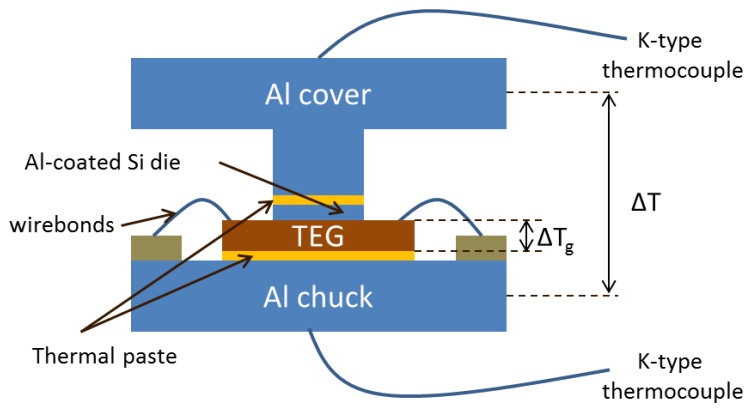
Schematic representation of the TEG in its housing.

The housing of the thermoelectric generator was designed to allow for the application of a macroscopic temperature difference across the hot and cold contacts of the thermocouples. This housing is schematically presented in Figure 2. The backside of the Si die was thermally connected to an Al chuck using thermal paste. The Al top metal layer was contacted by an Al-coated Si die, the back side of which was thermally connected through thermal paste to an Al cover (see Figure 2). The role of the Al-coated Si die was to protect the generator from scratching by the Al cover. A macroscopic temperature difference *ΔT* was applied between the Al chuck and the Al cover. In order to calculate the temperature difference *ΔT_g_* across the generator from the macroscopically applied *Δτ* we followed the standard reasoning in the literature [5,6,11] (ignoring Joule heating and peltier cooling in the generator because of no electrical current applied (open circuit)). The expression used was:

$$\Delta T_{g}=\frac{K_{g}}{K_{g}+K_{\text{top}}+K_{\text{bottom}}}\Delta T$$

where *K_g_* is the thermal resistance of the generator and *K_top_*, *K_bottom_* are the thermal resistances of the top and bottom structures used for the housing. The dimensions of the top Al cover were 1 cm × 10 cm × 10 cm for the large top plate and 1 cm × 1 cm × 1 cm for the smaller plate (protrusion) connected to the Si die. The dimensions of the Al coated Si die were 380 μm × 7 mm × 9 mm, while those of the bottom Al chuck were 1 cm × 10 cm × 10 cm. The average thickness of the thermal paste was considered to be 50 μm, as in other similar papers. Since the thermal resistance of the thermal paste accounts only for less than 10% of the total thermal resistance of both top and bottom metal parts of the housing, deviations from this value of thickness will not change significantly the results of the following discussion. Considering that the thermal conductivity of Al is 237 W/mK [5], that of the thermal paste is 1.4 W/mK (manufactor), that of Si is 149 W/mK [30], the thermal resistance of an Al-Al pressure contact is 9 K/W [5,11] for 1cm^2^ we get *K_top_* = 10.04 K/W and *K_bottom_* = 0.37 K/W. We have ignored all contact thermal resistances between the thermal paste and Al or Si pieces, as we believe they are negligible. For the generator itself *K_g_* is 0.65 K/W in the case of the 25 μm thick membranes and 1.22 K/W in the case of the 50 μm as described before and so we have:

Δ*T_g_* = 0.058Δ*T* in the case of the 25 μm thick porous Si membranes
Δ*T_g_* = 0.104Δ*T* in the case of the 50 μm thick porous Si membranes

In the above calculations we have assumed that *ΔT* is applied between the surface of the top Al cover plate and the backside of the Al chuck. Since the thermal resistance of the top Al cover plate and that of the Al chuck (equal dimensions with the Al top cover plate) are both 0.004 K/W, it is not important on which exact point of these two large Al plates of the housing the measurement of the temperature is performed.

The above 5.8% and 10.4% percentages of the macroscopic temperature difference found across the generator are in the same range as typical values reported in the literature (3.84% in [6] and 14.3% in [5]).

## 3. Fabrication

**Figure 3.**
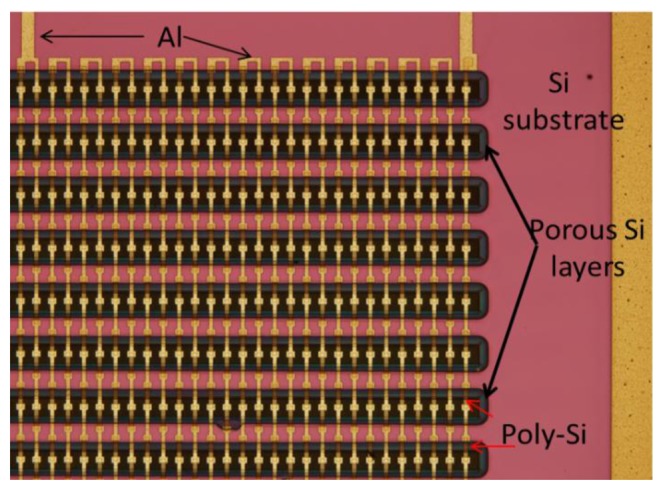
Optical microscope image of a part of the fabricated TEG without the top photoresist and Al layers.

The starting wafer for the fabrication of the TEG was p-type Si with resistivity 6–8 Ωcm. The fabrication process involves the following steps:

(a) Porous Si area formation. This is done by electrochemical dissolution of Si through an SiO_2_/poly-Si mask, as described in detail in [26]. The anodization is done in an HF/ethanol solution under a constant current density of 80 mA/cm^2^.
(b) Thermocouple formation. A 300 nm thick SiO_2_ layer is deposited on top of the entire substrate for electrical isolation and a 500 nm thick polysilicon layer is deposited by low pressure chemical vapor deposition, boron-doped at a dose of 10^16^ cm^−2^ at 60 keV and annealed at 1,050 °C for 30 min and patterned using lithography and etching. An Al layer, 500 nm thick, is then deposited on top and patterned to form the second metal lines of the thermocouples and the contacts. A detailed description of a similar fabrication process can be found in [24–26].
(c) Thermal isolation of the cold contacts of the thermocouples. A 350 nm thick SiO_2_ layer is first deposited by RF magnetron sputtering in order to provide the necessary electrical isolation. A layer of 1.6 μm thick photoresist (AZ5214E, MicroChemicals, Ulm, Germany) is then spun on and patterned in order to provide thermal isolation to the cold contacts of the thermocouples, with windows on top of the hot contacts. A 2 μm thick Al layer is then deposited on top of the structure and finally a lithography and etching step is applied for defining the access to the contact pads. An optical microscope image of a top view of the TEG without the photoresist and the top Al layer is shown in Figure 3.

Two different TEGs were processed and characterized. The first batch was composed of TEGs with 25 μm thick porous Si areas and a number of ∼668 thermocouples, covering a total area of 0.027 cm^2^. In the next, these devices will be referred to as TEG1 devices. The second batch was composed of TEGs with 50 μm thick porous Si areas and a number of ∼1,347 thermocouples, covering a total area of 0.053 cm^2^. They will be referred to in the next as TEG2 devices.

The above process for TEG fabrication is simple, it uses batch Si processing apart from the electrochemistry step for porous Si formation, and it does not contain any suspended part. It is thus robust and mechanically stable. The used porous Si layer provides thermal isolation of the hot contacts of the thermocouples, which is almost as efficient as in the case of the suspended membranes [23], with the additional advantages of robustness and batch Si processing. Moreover, compared to the TEGs using suspended membranes [5,6] the above TEG process leaves the backside of the wafer intact and planar so that there is no need to bond a second Si wafer on the backwside in order to assure good thermal contact and mechanical stability, as is the case in TEGs using suspended membranes [5,6].

## 4. Characterization and Discussion

**Figure 4.**
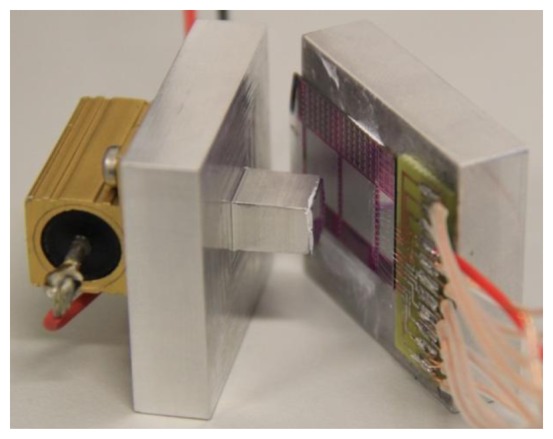
Picture of the measurement set up used for the characterization of the thermoelectric generator, depicting the top Al bath metal with the external resistor attached on it and the Al-coated TEG die.

For the testing of the TEG, a heating power was applied on the Al cover using an external resistor, as illustrated in Figure 4. The Al chuck at the backside of the TEG was naturally cooled by the ambient air. For practical applications, natural cooling is preferred to the Peltier cooling, used in most reports in the literature [5,6,10]. The temperature on both the top Al bath metal and the Al chuck was monitored using commercial K-type thermocouples. In order to make the measurement, the Al plates are positioned as shown in Figure 4 with the resistor fixed against a stable point. The part with the generator is then pushed towards the top part by using the pressure applied by one finger. The output voltage reaches a maximum with very little pressure applied and increasing the pressure does not cause any measurable difference.

The internal resistance of the generator was measured by a multimeter and found to be *R_g_* = 0.16 MΩ in the case of TEG1 and *R_g_* = 0.33 MΩ in the case of TEG2. The above values correspond to the expected ones from Equation (3) by considering the dimensions of the thermocouples and the resistivity of the materials used (see Table 1). This means that the contact resistance between the different metals is negligible.

**Figure 5.**
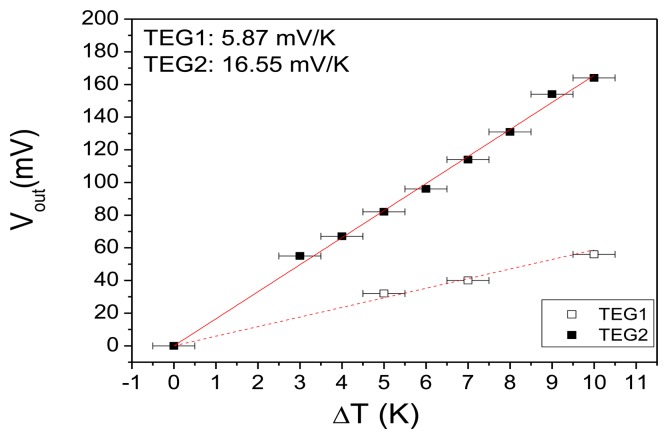
Output voltage (*V_out_*) as a function of the macroscopic temperature difference across the device (*ΔT*) for TEG1 and TEG2. The red lines represent linear fits to the experimental data.

The output voltage (*V_out_*) of TEG1 and TEG2 as a function of the macroscopic temperature difference (*ΔT*) is shown in Figure 5. The temperature of the Al chuck (cold contact) was between 20 and 23 °C for the measured *ΔT*. The response is linear for both devices and the value of the slope is 5.87 mV/K for TEG1 and 16.55 mV/K for TEG2. The difference between the two types of TEGs is the number of thermocouples and the thickness of the porous Si layer. We emphasize again that these measurements were obtained with the reference temperature (cold part of the thermocouples) at ambient temperature, without any additional cooling. The testing conditions are thus closer to practical applications. The actual temperature difference across the thermoelectric generator (*ΔT_g_*) can be calculated from the macroscopically measured temperature difference by considering the temperature differential evaluated in the design section, namely that only 5.8% of the macroscopically applied temperature difference *ΔT* in TEG1 and 10.4% of *ΔT* in TEG2 reaches the thermocouples. By considering the measured output voltage of each device against the actual temperature difference *ΔT_g_* across the TEG and the number of thermocouples in each device, the value of the Seebeck coefficient of the poly-Si material can be extracted. The extracted value for TEG1 is 151 μV/K, which is very close to the literature value of poly-Si Seebeck coefficient (see Table 1). This provides a large degree of confidence in the calculation of *ΔT_g_*.

**Figure 6.**
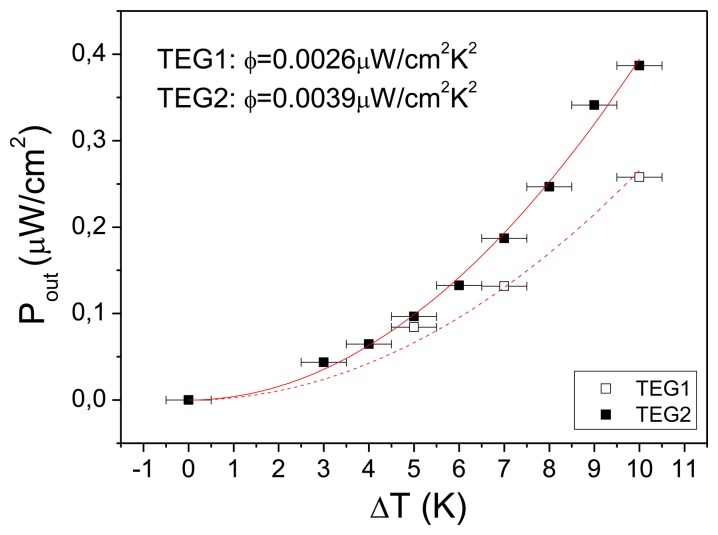
Output power (*P_out_*) as a function of the macroscopic temperature difference across the device (*ΔT*) for TEG1 and TEG2. The red lines represent parabolic fits to the experimental data.

The results for the output power at matching load are presented for both TEG1 and TEG2 in Figure 6. TEG1 (projected to 1 cm^2^) can produce 0.26 μW at a temperature difference of 10 K while TEG2 (also projected to 1 cm^2^) can produce 0.39 μW for the same temperature difference. The above values compare advantageously with the values in the literature of 0.23 μW, projected to a 10 K temperature difference, in [13] and 0.25 μW for a 10 K temperature difference but an area smaller than 1 cm^2^ in [9].

The power efficiency factors for TEG1 and TEG2 were found to be *φ* = 0.0026 μW/cm^2^·K^2^ and *φ* = 0.0039 μW/cm^2^·K^2^, respectively. These values are the actual measured values for the macroscopically applied temperature difference with the cold part of the measurement set-up not forced to a certain temperature. These results are therefore very similar to what could be achieved in a practical application. Efficiency factors in the literature are usually calculated considering the calculated temperature difference across the generator (*ΔT_g_*). Using this assumption the power efficiency factor of devices fabricated by MEMS techniques in the literature are 0.016 μW/cm^2^·K^2^ in [5], 0.052 μW/cm^2^·K^2^ in [6], 0.026 μW/cm^2^·K^2^ in [7], 0.0426 μW/cm^2^·K^2^ in [10], 0.0417 μW/cm^2^·K^2^ in [11] and 0.000064 μW/cm^2^·K^2^ in [12]. If we consider the output power per cm^2^ of TEG2 as a function of *ΔT_g_* a power efficiency factor of 0.365 μW/cm^2^·K^2^ is calculated. This is because, as shown before, *ΔT_g_* = 10.4%ΔT. The theoretical value for the power efficiency factor can be calculated considering the values of the Seebeck coefficient, the resistivity (see Table 1), the number of thermocouples and the area covered by the thermocouples. For the thermoelectric generator presented in this work this theoretical value is 0.56 μW/cm^2^·K^2^. The value calculated from the measurements is clearly very close to this theoretical value. This 35% difference between the theoretical and calculated value from the measurements could be explained by the simplicity of the estimates in the calculation of *ΔT*_g_. By ignoring the contributions of thermal resistance of all areas with photoresist on top of porous Si, as well as the contributions of all thermal contacts except the Al-Al one, we have underestimated the percentage of *ΔT* that is translated to *ΔT_g_*. Also, fabrication imperfections such as photoresist walls in the openings not perfectly vertical or small fluctuations in the photoresist thickness or even slight misalignment of the openings in the photoresist and the porous Si layers can also affect the experimental result. Furthermore in the calculation of the power efficiency factor the contribution of the temperature differential is squared and so the previously mentioned sources of error are magnified.

The above results prove that an efficient thermoelectric generator can be fabricated based on the thermal isolation on the Si wafer provided by a thick porous Si layer. In this work, a single 1.6 μm thick photoresist layer was used to provide thermal isolation between the hot and cold contacts of the thermocouples. Thicker layers of top thermal isolation can increase the thermal resistance of the generator, thus enabling the increase of *ΔT_g_* for a given *ΔT*. This, in turn, can increase the produced output voltage and power for a given *ΔT*. Further improvement can be achieved by optimizing the thermocouple materials, by using for example p-doped/n-doped poly and material nanostructuring for increasing the ZT [17]. By gaining a factor of 2 in the output power we can reach the 1 μW/cm^2^ at 10 K, which is the goal for rendering TEGs useful for practical applications [10].

## 5. Conclusions

A novel thermoelectric generator based on porous Si thermal isolation on the Si wafer and p-doped poly-Si/Al thermocouples was designed, fabricated and tested. The fabrication process uses standard Si batch processing steps and a single electrochemistry step. The device does not contain any suspended parts; it is thus more robust than MEMS thermoelectric devices. The testing of the TEG showed that with the 50 μm thick porous Si layer an output power of 0.39 μW/cm^2^ for a macroscopic temperature differential of 10 K is achieved. By decreasing the thickness of the porous Si layer to 25 μm, the TEG performance is deteriorated. The measured output power of the presented TEG exceeds reports in the literature under similar measurement conditions. The excellent output characteristics of the generator, combined with the simplicity of its fabrication process, make it a promising candidate for practical applications.

## Figures and Tables

**Table 1.** Thermal and electrical properties of the materials used for the design of the thermocouples.

**Material**	**Seebeck Coefficient (μV/K)**	**Thermal Conductivity (W/mK)**	**Electrical Resistivity (Ω.m)**
p-doped poly-Si	147 [6]	31.4 [6]	(2.2 ± 0.2) × 10^−5^ [measured]
Al	−1.7 [5]	237 [5]	4.3 × 10^−8^ [5]
